# An open-source deep learning model for classifying real-world activity intensities in persons with chronic pain using a wrist-worn accelerometer

**DOI:** 10.3389/fdgth.2026.1783507

**Published:** 2026-10-14

**Authors:** Annet Doomen, Richard Felius, Harriët Wittink, Ivan Huijnen, Michiel Punt, Rob Smeets

**Affiliations:** 1 Department Healthy and Sustainable Living, University of Applied Sciences Utrecht, Utrecht, Netherlands; 2 Department of Rehabilitation Medicine, Research School CAPHRI, Maastricht University, Maastricht, Netherlands; 3 Adelante Centre of Expertise in Rehabilitation and Audiology, Hoensbroek, Netherlands; 4 Research Centre on Appropriate Rehabilitation Care, Zuyd University of Applied Sciences, Heerlen, Netherlands; 5 CIR Clinics in Rehabilitation, Eindhoven, Netherlands; 6 Pain in Motion International Research Group (PiM), Brussels, Belgium

**Keywords:** accuracy, IMU, machine learning, persistent pain, sensors

## Abstract

**Introduction:** In chronic pain (CP) care, patterns of activity and rest are relevant across diagnostic, intervention, and evaluation phases. Objective measurement of these patterns can support individualized care. Wrist-worn tri-axial accelerometers are user-friendly and yield valid and reliable data in the general population. Public availability of a detailed dataset together with a validated deep learning (DL) model for classifying activity intensities from raw accelerometer data can promote clinical use and standardize activity intensity measurement in CP research.

**Methods:** This study aimed to (1) develop, test, and share a DL model that classifies activity intensity from raw accelerometer data, (2) explore whether CP-specific training improves performance in CP, and (3) explore construct validity in real-world settings among individuals with and without CP, using real-world observations as comparator instrument. Seven strategies were applied to train a Convolutional Neural Network, using three datasets comprising 211,864 ten-second windows. Construct validity was evaluated using 3,443 one-minute real-world observations from 5 individuals with and 10 without CP.

**Results:** Classification accuracy was 76.5% in individuals with CP and 80.5% in individuals without CP. There was no clear advantage of CP-specific training. Real-world agreement was 73.6% in CP and 78.6% in individuals without CP.

**Discussion:** Lower and individually variable real-world agreement, particularly in CP, indicates that model performance may depend on individual characteristics and the comparator instrument. The results suggest that the accompanying script and datasets enable accurate processing of raw accelerometer time series to activity intensities in the CP populations studied. Further investigation of construct validity and other clinimetric properties is warranted.

## 1 Introduction

Chronic Pain (CP) has a lifetime prevalence of 12.7%–33.7% in Europe (1) and is defined as pain that recurs or lasts for at least 3 months (2). Persons with CP report that their pain significantly interferes with many aspects of daily life (3). To cope with both their pain and daily demands, they often adapt their patterns of activity and rest.

An activity pattern refers to the succession of rest and activity, and is defined as the temporal structure of physical activity and sedentary behavior accumulated over a specified period during waking hours (4). These patterns are considered clinically relevant because they may reflect, and help explain, psychological state, physical function, and pain interference (5–8).

Within CP populations, pain-coping strategies can influence activity patterns (9). Strategies include fear-avoidance, excessive overexertion, and pacing. In fear-avoidance, fear of activity-related pain ultimately leads to avoidance of activities (10). Excessive overexertion is characterized by a “sawtooth” pattern of activity intensity and pain: individuals engage in activity despite increasing pain, followed by pain-induced collapse and prolonged rest (11). This strategy may stem from a perceived need to maintain a desired self-identity (e.g., “I cannot give up”). Although debated (12, 13), both strategies are generally considered maladaptive, as they may contribute to increased symptom burden, physical deconditioning, limitations in activities and participation, depression and higher susceptibility to pain (11, 14). In contrast, pacing is regarded as an adaptive strategy. It involves regulating activity and rest by, amongst others, breaking down tasks, finding baselines of tolerable activities, planning activities in a time-contingent manner and setting goals (15). The principles underlying these activity-related coping strategies are applied in CP care to improve daily functioning and participation (6, 15–17).

Indicators for different activity patterns can be extracted from activity monitors, like accelerometers. Accelerometer data can be converted into time series of activity intensities, from which activity-pattern indicators can be derived (18, 19). However, activity monitors are not yet routinely employed in CP rehabilitation. Instead, activity patterns are typically assessed through self-reported questionnaires or reported by patients during interviews by healthcare providers. As such, these patient-reported outcomes rely on recall of activity behavior, reference values and perceptions, while activity monitors provide information on actual activity behavior, and self-reported activity has been shown to correlate weakly with activity monitor outcomes (20–22). Therefore, self-reported questionnaires, estimates and activity monitors are complementary, and the use of accelerometers may enhance accuracy and effectiveness of the intervention tailoring, and the monitoring of activity behavior.

Accelerometers have proven to be reliable and feasible for use in daily life (23, 24). Traditionally, raw accelerometer data have been translated into activity intensity using cut-off point thresholds or regression-based approaches (25). Recent advancements, however, suggest that deep learning (DL) models may outperform these conventional methods (23, 26–29), but empirical studies directly comparing cut-off points methods with DL are scarce (26, 27, 30). DL models offer high accuracy, adaptability across populations and settings, and the potential for continuous improvement with the addition of new data. However, two knowledge gaps currently limit the substantiated use of DL-based accelerometry in CP care.

First, the clinimetric properties of DL models have primarily been investigated in laboratory settings and their performance under real-world conditions remains underexplored (23, 31), leaving a gap in our understanding of their construct validity outside controlled environments. Second, it has been extensively theorized, and observed to some extent, that pain results in adapted motor behavior, ranging from subtle changes like increased stiffness or guarding, to avoidance of painful movements (32–37). These changes may alter accelerometer signals, which could reduce the accuracy of DL models trained on data from non-chronic pain populations in classifying activity intensities. To date, no studies have investigated whether DL models require adaptation to CP-specific motor behavior to maintain sufficient accuracy.

This study aims to address these gaps by (1) developing a DL model to classify activity intensities from accelerometer data collected in both laboratory and real-world settings in individuals with and without CP, (2) exploring whether CP-specific training enhances DL model performance for individuals with CP relative to training using data from individuals without CP and from the general population, and (3) exploring the model's construct validity by comparing the output with observations in real-world conditions.

The datasets and the model script are available as open-source resources to facilitate direct application to raw triaxial accelerometer data from CP populations.

A DL model capable of accurately converting accelerometer signals to activity intensity levels in real-world settings for individuals with CP could enhance personalized care by improving the assessment and evaluation of activity behavior.

## 2 Materials and methods

### 2.1 Populations

The research protocol was approved by the Ethical Committee Research Healthcare Domain (ECO-GD) of HU University of Applied Sciences Utrecht (reference 193-000-2022).

#### 2.1.1 DL model development

For DL model development, raw accelerometer datasets from three different populations were used. The first two datasets are referred to as Lab data, whereas the third dataset is referred to as Capture data.

First, 30 participants without CP (healthy participants, HP; “HP lab data”) were recruited from the researchers' network and via advertisement in the newsletter of University of Applied Sciences Utrecht (Institute of Movement Studies). Inclusion criteria were: at least 18 years of age, no CP, and not having any illness, disease or mobility aid use that substantially affects movement. This latter criterion was added because significant deviations from normal movement, like pronounced asymmetric movement, spasms or using crutches, might interfere with activity intensity recognition. HP were purposively selected to ensure alignment as closely as possible with the dataset of participants with CP.

Second, 30 participants with CP (“CP lab data”), who had started interdisciplinary pain rehabilitation at CIR rehabilitation clinics Eindhoven, The Netherlands and at Adelante rehabilitation center, Hoensbroek, The Netherlands, were included by their care providers. Inclusion criteria were having movement-evoked CP (like primary CP, secondary musculoskeletal CP, and neuropathic CP), with limitations in daily activities and/or participation, and other criteria coincided with the HP inclusion criteria. Participants with CP were rewarded with a €25 gift certificate.

Third, we implemented the Capture-24 dataset. This dataset is a large open-source dataset of annotated real-world accelerometer data (38). It became available during processing of the first two datasets and it was added to improve stability of the DL model. For this dataset 151 participants were recruited from the general population of Oxfordshire, United Kingdom, following advertisement, via professional networks, posters, social and sport clubs, word of mouth, and research volunteers by market research agency. Inclusion criterion was age 18 and older, and purposive sampling was applied to guarantee a sample across age, sex and educational level (39).

#### 2.1.2 Real-world construct validity

For investigating construct validity in real world, 10 HP were recruited from the researchers’ network. Participants were purposively selected to yield a variety of age, sex and body mass index (BMI). Moreover, 5 CP were recruited through convenience sampling from the researchers' network, advertisements in the newsletter of University of Applied Sciences Utrecht (Institute of Movement Studies), and social media. These 10 HP and 5 CP were not involved in DL model development. Inclusion criteria for HP and CP corresponded to those of DL model development. Figure 1 provides an overview of the different datasets included in this study.

**Figure 1:**
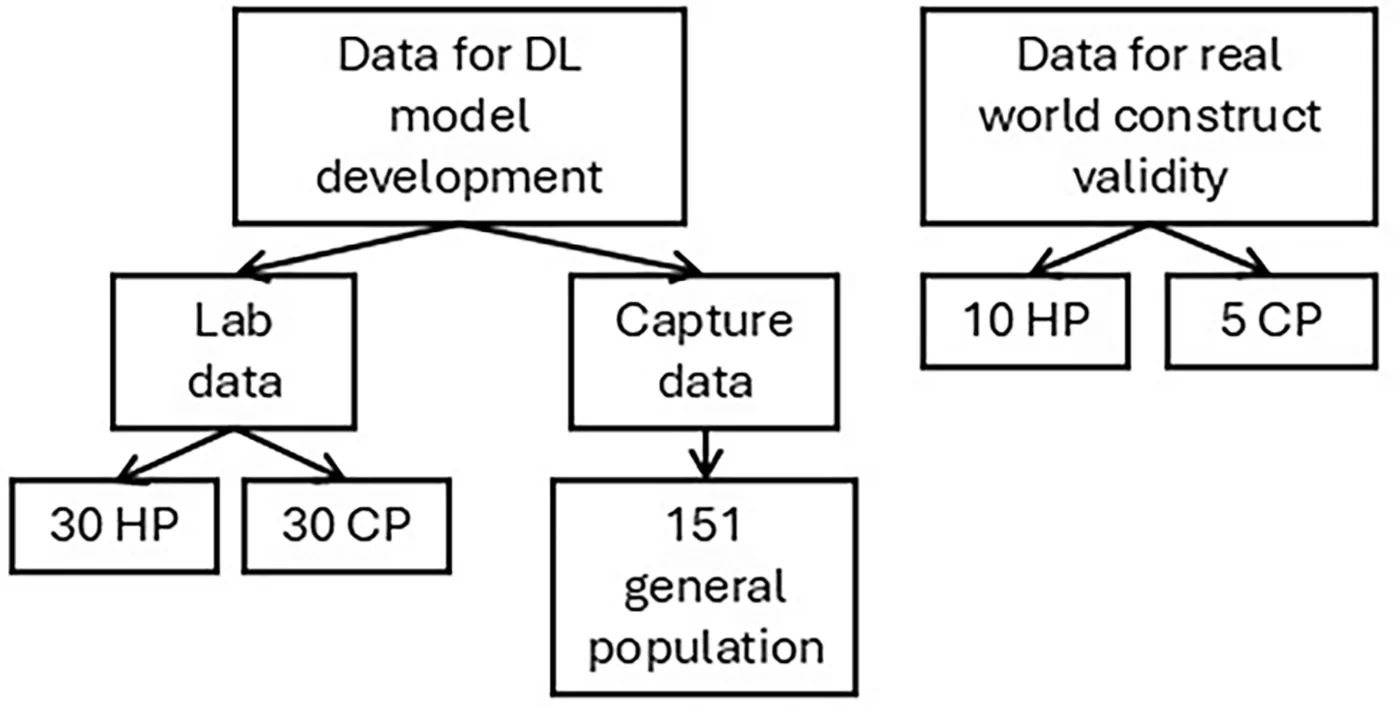
Overview of datasets used for DL model development and for real-world construct validity.

### 2.2 Data collection

#### 2.2.1 Triaxial accelerometer

For Lab data and construct validity a triaxial accelerometer (Aemics B.V., Oldenzaal, the Netherlands) was used, which sampled at 12.5 Hz, and stored the recordings on an internal memory card. This relatively low frequency was selected to optimally capture the complexity of human movement, as higher frequencies have not shown improved performance (40–42). The accelerometer was worn on the lateral side of the dominant wrist, based on evidence that DL performance is largely independent of sensor placement (26, 40, 43–45), wrist-worn sensors yield higher adherence than hip-worn devices (46), and dominant wrist placement may offer greater accuracy (47, 48).

For Capture data an Axivity AX3 tri-axial accelerometer was employed, with comparable specifications to the Aemics device. Participants wore the accelerometer on their dominant wrist. The sampling frequency was set at 100 Hz. This discrepancy with Lab data settings was addressed during data processing.

#### 2.2.2 Data collection for DL model training, testing and validation

HP lab data were collected in a semi-controlled laboratory setting at HU University of Applied Sciences, Utrecht, The Netherlands, and for CP participants at their rehabilitation center. All participants received oral and written information and provided informed consent.

HP and CP participants were instructed to perform 21 predefined activities (Table 1), each lasting 80 s, while wearing the accelerometer. Activities were supported with appropriate furniture and tools and conducted in a private room. Participants could opt out of any activity if they felt unsure, incapable or uncomfortable. Cycling was performed outdoors on a utility bicycle; walking and running took place in a corridor with a bend; stair climbing occurred in a stairwell, with repetitions adjusted to meet the 80-second duration. Activity start and end were indicated by the researcher (AD for HP, and a physiotherapist and a physiotherapy trainee for CP participants), and participants marked these moments with three rapid forearm shakes, producing distinct acceleration peaks for segmentation. Each activity bout was labelled with an activity identifier.

**Table 1:** Twenty-one pre-defined activities performed in a semi-controlled setting, their MET-level (49) and activity intensity class.

Activity	MET-level (kcal/kg/h)	Activity intensity class
Sitting reading a book	1	Sedentary
Sitting reading a newspaper	1	
Sitting having a drink	1.3	
Sitting talking	1.3	
Sitting checking phone	1.3	
Lying down reading a book	1	
Lying down watching TV	1	
Lying down checking phone	1	
Lying down talking to someone	1	
Sitting desk work	1.5	
Walking slow pace (3 km/h)	2	2. Light intensity
Standing cooking	2.7	
Standing talking	1.8	
Standing still	1.5	
Standing in the cold	1.7	
Walking fast pace (5.3 km/h)	3.8	3. Moderate-to- vigorous intensity
Standing doing do-it-yourself tasks	4.0	
Stair climbing slow pace	4.5	
Descending stairs	3.5	
Running easy pace	7.8	
Cycling as to/from work, self-selected pace	6.8	

For Capture data, participants wore an Axivity and a neck-worn camera for approximately 24 h in free-living conditions. Time-stamped camera images were used to annotate the accelerometer data (38). Sleep data were excluded from our analysis.

The activities of Lab data and Capture data were classified to level of Metabolic Equivalent of Task (MET; kcal/kg/hour) with the Adult Compendium of Physical Activities 2024 (49) and 2011 (50), respectively. For Capture data, 74 activity codes from the Adult Compendium were used (38). The MET-levels were classified to activity intensity categories 'sedentary' [MET ≤ 1.5, while in sitting, reclining or lying position (51)], “light intensity” (1.5 < MET ≤ 3), “moderate-to-vigorous intensity” (Moderate to Vigorous Physical Activity; MVPA; MET > 3) (Table 1). Classification from MET to activity intensity class was not completely consistent in the Capture data as 15 light intensity activities were classified as sedentary and 4 MVPA activities were classified as light intensity, which can be deduced from the annotation table that is included in the GitHub repository accompanying the paper of Chan et al. (38). We adjusted this classification before training our DL model.

Relative to the Adult Compendium of Physical Activities, some adjustments were made to the MET-levels assigned to the HP and CP Lab data activities. “Cooking while standing” was assigned MET 2.7, reflecting the average of “Cooking moderate effort” (MET 3.5) and “Cooking light effort” (MET 2.0). “Standing still” was assigned MET 1.5 instead of 1.3, and classified as light intensity as standing is metabolically not considered sedentary (52), and “Standing in the cold” was estimated at MET 1.7. “Standing doing do-it-yourself tasks” was assigned MET 4.0, based on the average of moderate effort carpentry (MET 4.3), moderate effort house painting (MET 3.3) and moderate effort home repair (MET 4.5).

#### 2.2.3 Real-world accelerometer data for construct validity

Construct validity was evaluated by comparing the output of the DL model with real-world observations. To this end, 10 HP and 5 CP participants were observed in their daily-life environments for approximately four hours while wearing the wrist-mounted accelerometer. Participants were instructed to follow their usual routines without modification.

The observers (a physiotherapy student, a psychosomatic physiotherapy student, a senior researcher, and AD) recorded activity descriptions every minute in an Excel spreadsheet. If multiple activities occurred within a minute, each was documented, along with its duration. Annotations and accelerometer data were synchronized by simultaneous starting and stopping of the accelerometer and the stopwatch.

### 2.3 DL model

#### 2.3.1 Data processing

HP and CP Lab data and Capture data were used to develop the DL model. For Lab data, activity intensity labels (sedentary, light, MVPA) were synchronized with the raw accelerometer data based on the predefined activity intensity labels. Forearm shake events were detected using vector magnitude calculations and a moving average filter to reduce noise, followed by a peak-finding algorithm. All files were visually inspected for accuracy. In cases of anomalies, such as inconsistent shake counts, two researchers (AD and RF) independently reviewed the data and resolved discrepancies through discussion. Unresolved cases were excluded.

The activity bouts were matched with the activity labels, and unlabeled data segments (representing transfer periods between two activities) were removed from the dataset. To minimize noise from transitions, the first and last four seconds of each labeled segment were excluded.

Data processing of the Capture data is described in Chan et al. (38). Capture data were down sampled to 12.5 Hz by preserving every eighth sample from the original data.

Lab data were segmented using a sliding window of 10 s with 50% overlap to increase the number of windows. Capture data were segmented in 10-second windows without overlap, as its volume was sufficient to support model development.

Outliers were identified for each activity by converting the mean and standard deviation of each acceleration axis (anteroposterior, mediolateral and vertical) into *z*-scores. Windows with *z*-scores exceeding five were classified as outliers and excluded from further analysis (53).

#### 2.3.2 Training, testing and validating the deep learning model

The labeled 10-second windows were used to train, test and validate four initial DL models for classifying activity intensities in three classes. Each model was trained, tested and validated with both Lab data and Capture data. In addition to these DL models, we developed a baseline cut-off points model using a Gaussian Mixture Model, and we examined the effect of downsampling Capture data on performance. Supplementary Appendix A provides descriptions of the architectures and performance metrics for all five models, along with a comparison of two models trained using 100 and 12.5 Hz Capture data.

From these four initial models, a convolutional neural network (CNN; accuracy 67.7%, *F*1 score 66.1%) was selected for its balance between performance and model size.

Subsequently, the CNN-model was trained, tested and validated with seven different strategies (Figure 2). This approach enabled evaluation of model performance across various populations. First, as a reference, the model was trained, tested and validated three times using data from a single source: Capture data (“Capture on Capture”), HP lab data (“HP on HP”) and CP lab data (“CP on CP”). Second, training and testing on HP and validating on CP (“HP on CP”) provides further information on whether CP need a CP-specific model. Third, to further assess consistency across populations, three models were trained and tested on the combined datasets and validated separately on each of the three sets (“All on Capture”, “All on HP” and “All on CP”).

**Figure 2:**
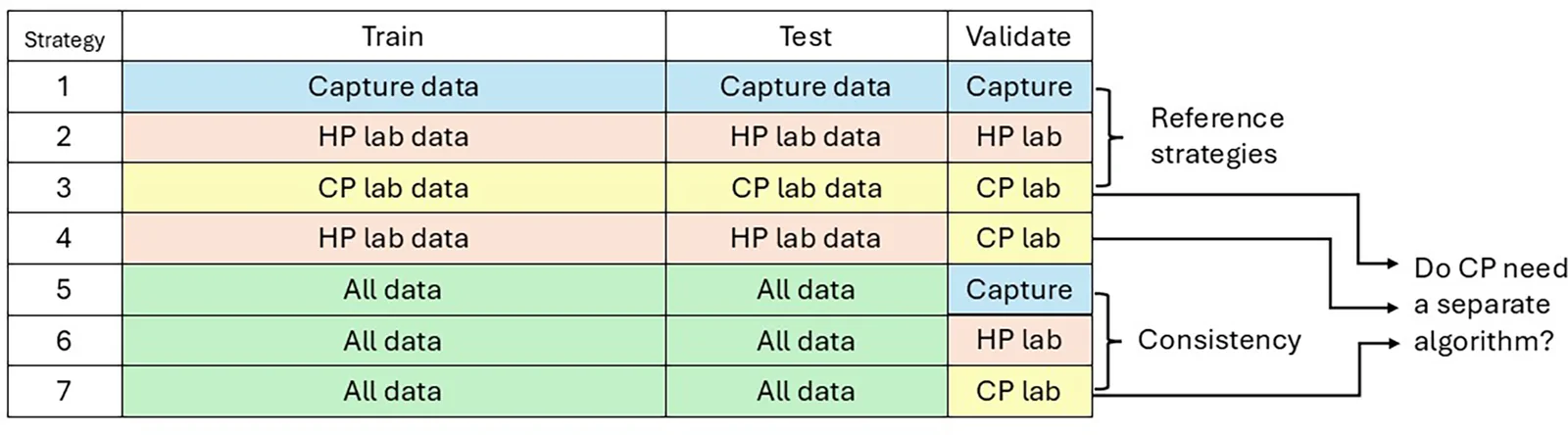
Structure and purpose of the seven training, testing and validation strategies.

To address class imbalance, category-specific weights were applied during training. The model was trained and evaluated with repeated 10-fold cross-validation, with data split at the subject level into training (∼70%), testing (∼20%) and validation (∼10%) sets. The train and test sets served for training and evaluating during the training phase, and the validation set served for model performance evaluation after training. Participants were randomly assigned to one of ten folds. In each iteration, one fold was used for validation, approximately two folds for testing, and the remaining folds for training. Each participant appeared exactly once in the validation set across the ten iterations. This procedure was repeated ten times.

The Python 3.12.7 scripts for data-processing, the DL model, and the data are publicly available on https://zenodo.org/records/17801499.

#### 2.3.3 Performance analysis and comparison

For each of the seven strategies, performance metrics—including accuracy, Macro-*F*1 score, recall and precision—were calculated per iteration. Accuracy reflects how often the model is right. *F*1 scores reflect how well the model finds and correctly identifies the classes of interest, and combines precision and recall. Precision is the proportion of positives that are truly positive, and recall is the proportion of actual positives that are correctly identified.

Mean, standard deviation and range were determined for each metric, per model. Additionally, confusion matrices were constructed to evaluate classification performance across the three activity intensity classes. Performance metrics in the range of 70%–90% are generally considered indicative of good model performance, suggesting that the model outputs are suitable for the use in subsequent analyses.

To determine whether a CP-specific model provided added value, the performance of the “CP on CP”, “HP on CP”, and “All on CP” strategies was compared descriptively.

### 2.4 Real-world construct validity

After conducting the observations of 10 HP and 5 CP participants in daily life settings, two of the four researchers independently assigned a MET value to each recorded activity for every participant. These values were derived from the 2024 Adult Compendium of Physical Activities (49). Prior to classification, the researchers completed a training session to ensure consistency. MET values were averaged per minute, compared between the two raters, and any discrepancies were discussed and resolved. Finally, the resulting MET values were categorized into one of three activity intensity classes.

To compare observer-based and DL-based classifications, the 10-second DL predictions were aggregated by summing the predicted probabilities for each class across the 10-second intervals within a minute and selecting the class with the highest total probability. Percentage of agreement was then calculated for each participant using the observer classifications as the comparator instrument. These calculations were repeated with ten model runs, and results were averaged per participant. Subsequently, group mean was calculated, and range and standard deviation were determined based on the participant-level mean agreement scores. We hypothesized substantial agreement between the two measures of at least 70% and a weighted Cohen's kappa (linear weights) of at least 0.61, which may be interpreted as substantial agreement (54). Confusion matrices further illustrated where errors occur.

## 3 Results

### 3.1 Descriptives

For DL model development, three different datasets were implemented. We recruited 30 individuals with CP and 30 HP, and the Capture dataset included 151 participants. Participant characteristics are summarized in Table 2. To examine whether individuals with CP and HP require separate DL models for accurate activity intensity classification, the groups ideally need to be comparable. Therefore, we attempted to match the 30 individuals with CP and the 30 HP on sex, age, BMI, and dexterity. Nevertheless, mean BMI and age were higher in the CP group. For all participants with CP, average pain intensity score during the previous week, pain duration, and the Pain Disability Index (PDI), were collected. For construct validity, five CP participants and ten HP were observed for approximately four hours.

**Table 2:** Participant characteristics of CP lab data, HP lab data, and capture data included for training and testing the CNN-model, and of HP and CP included for construct validity.

	CP lab data (*n* = 30)	HP lab data (*n* = 30)	Capture data (*n* = 151)	Construct validity CP (*n* = 5)	Construct validity HP (*n* = 10)
Sex (female/male)	19/11	19/11	99/52	5/0	7/3
	Average (sd), range	Average (sd), range	Average, %	Average (sd), range	Average (sd), range
Age (years)	49.3 (13.7), 23–73	42.3 (14.1), 21–66	18–29: 43 (28.5%)	66.6 (12.0), 44–78	43.5 (14.1), 24–68
			30–37: 37 (24.5%)		
			38–52: 37 (24.5%)		
			≥53: 34 (22.5%)		
Length (cm)	173.2 (10.0), 157–195	173.1 (9.3), 150–199	N.R.	168.0 (7.0), 160–178	174 (8.0), 161–187
BMI (kg/m^2^)	27.5 (7.0), 17.8–44.3	23.9 (2.9), 19.4–33.0	N.R.	26.2 (4.4), 21.1–32.3	27.68 (9.1), 18.8–43.6
Dexterity (right/left)	28/2	28/2	N.R.	5/0	9/1
Pain intensity (0–10)	6.4 (1.4), 3–9	N.A.	N.A.	3.6 (1.7), 1–6	N.A.
Pain duration (years: *n*)	0.25–1: 9	N.A.	N.A.	2–5: 2	N.A.
	1–2: 3			>5: 3	
	2–5: 4				
	>5: 14				
CP Diagnosis (*n*)	Fibromyalgia: 14	N.A.	N.A.	CPTP: 1	N.A.
	Generalized pain: 5			Specific LBP: 1	
	Osteoarthritis: 5			Neuropathic pain: 2	
	NSLBP: 4			CPSP: 1	
	Neuropathic pain: 2			Fibromyalgia: 2	
	CPSP: 2			PFPS: 1	
	CPTP: 4				
	Post-viral pain: 1				
Pain disability (PDI^a^, 0–70)	41.5 (9.7), 22–61	N.A.	N.A.	21.6 (8.3), 11–32	N.A.

N.R., not reported; N.A., not applicable; NSLBP, non-specific low back pain; CPSP, chronic post-surgical pain; CPTP, chronic post-traumatic pain; LBP, low back pain; PFPS, patellofemoral pain syndrome. Participants could have multiple diagnoses.

a PDI, Pain Disability Index, 0–70, 0 = no disability, 0–10: minimal impact on daily activities, 11–30, mild disability, 31–50: moderate disability, 51–70 severe disability.

For the CP lab data the 21 activity intensity labels of three CP participants could not be matched with the acceleration data because of inconsistencies between the recorded bout lengths per activity and the bout lengths in the raw accelerometer data. These three participants with CP were excluded. Two of these participants were male, all three were right-handed, their average age was 58 years, BMI 31.0 kg/m^2^, pain intensity 6.3, and pain disability 38.

The training data from the remaining 208 participants contained a total of 211,864 10-second windows (588 h; 35,280 min) of which 15,159 windows (7.2%) originated from CP and HP lab data. A total of 549 windows (0.5%) were identified as outliers and excluded from further analysis.

The construct validity data contained 2,436 real-world observation minutes for HP and 1,007 min for CP. The distribution of activity intensities in the different datasets is illustrated in Figure 3.

**Figure 3:**
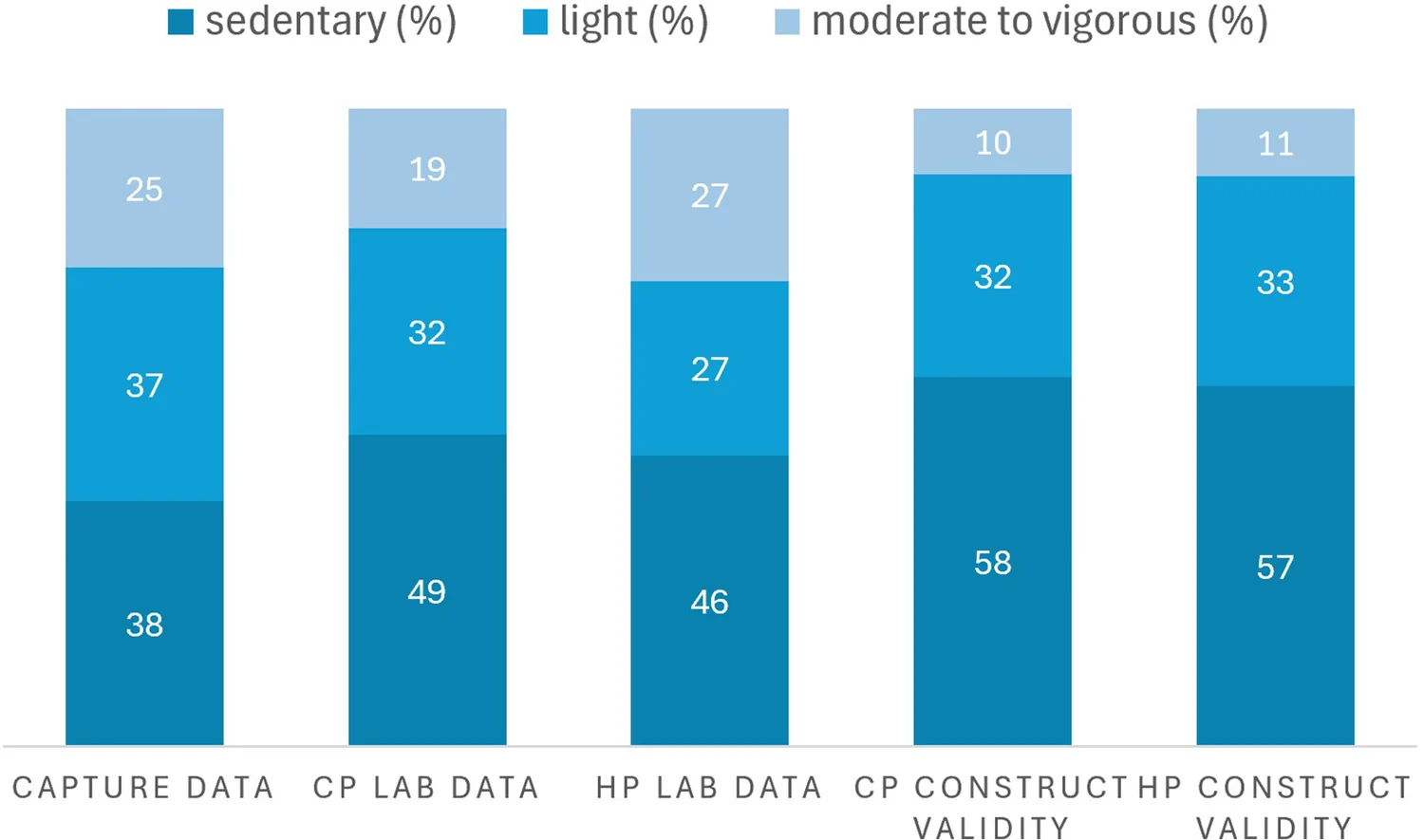
Distribution of training data and of observation minutes for construct validity, across sedentary, light and MVPA intensity activities, in persons without pain (HP) and with chronic pain (CP).

### 3.2 Performance of the CNN model

The DL model was trained, tested and validated with seven strategies. The corresponding performance metrics are presented in Table 3. Accuracy ranged from 67.2% to 81.3% and Macro-*F*1 scores ranged from 66.1% to 79.7%. The highest performance was achieved with the “HP on HP” strategy, whereas the strategies validated on Capture data (“All on Capture” and “Capture on Capture”) yielded the lowest scores. Additionally, strategies validating on CP lab data yielded lower performance than those validated on HP lab data.

**Table 3:** CNN performance values of activity intensity classification across the three intensity classes, for each of the seven strategies.

CNN-model	Accuracy	Macro-*F*1 score	Recall	Precision
All on CP (SD) [range]	76.5 (3.6), [68.9, 82.9]	73.4 (4.2), [62.4, 81.2]	72.8 (4.2), [60.8, 81.2]	75.2 (4.4), [66.3, 83.1]
All on HP (SD) [range]	80.5 (3.7), [71.3, 88.6]	78.3 (4.2), [68.0, 87.2]	77.7 (4.4), [67.3, 87.2]	80.1 (3.7), [69.0, 87.3]
All on Capture (SD) [range]	67.2 (1.9), [63.7, 71.4]	66.1 (1.8), [62.7, 70.2]	65.7 (1.8), [61.8, 69.7]	68.0 (1.6), [65.4, 72.4]
HP on CP (SD) [range]	77.9 (3.6), [71.7, 85.7]	75.1 (4.2), [67.8, 83.3]	74.7 (4.1), [67.6, 82.8]	76.8 (4.5), [67.9, 86.4]
CP on CP (SD) [range]	77.1 (5.6), [60.8, 88.4]	73.9 (6.4), [57.3, 87.0]	74.9 (6.2), [58.6, 86.9]	75.6 (5.8), [57.2, 87.3]
HP on HP (SD) [range]	81.3 (5.6), [62.3, 91.7]	79.7 (5.8), [61.1, 91.3]	79.6 (5.6), [60.8, 91.2]	81.4 (5.0), [66.3, 91.3]
Capture on Capture (SD) [range]	67.4 (1.8), [62.4, 71.1]	66.2 (1.8), [61.7, 70.2]	65.9 (1.8), [62.0, 69.7]	67.9 (1.8), [62.6, 71.3]

Notably, strategies incorporating Capture data showed lower standard deviations and narrower performance ranges compared to those without Capture data. This suggests that the inclusion of Capture data enhances the stability of the DL model's output. Additionally, downsampling the Capture data appeared to decrease accuracy from 71.8% to 67.4% (Supplementary Appendix A). This decrease was considered acceptable given its relatively small magnitude compared to the larger performance gains achieved by incorporating Lab data (Table 3).

As depicted in Figure 4, in CP, sedentary intensity was correctly classified in 89% of cases, light intensity in 64% and MVPA in 66%. Across all strategies, both MVPA and light intensity were more frequently underestimated than overestimated. This trend was particularly pronounced in strategies that included Capture data. In CP, light intensity activity was underestimated as sedentary in 25% and MVPA was underestimated as light intensity in 29%. In HP these percentages were 26% and 22% respectively.

**Figure 4:**
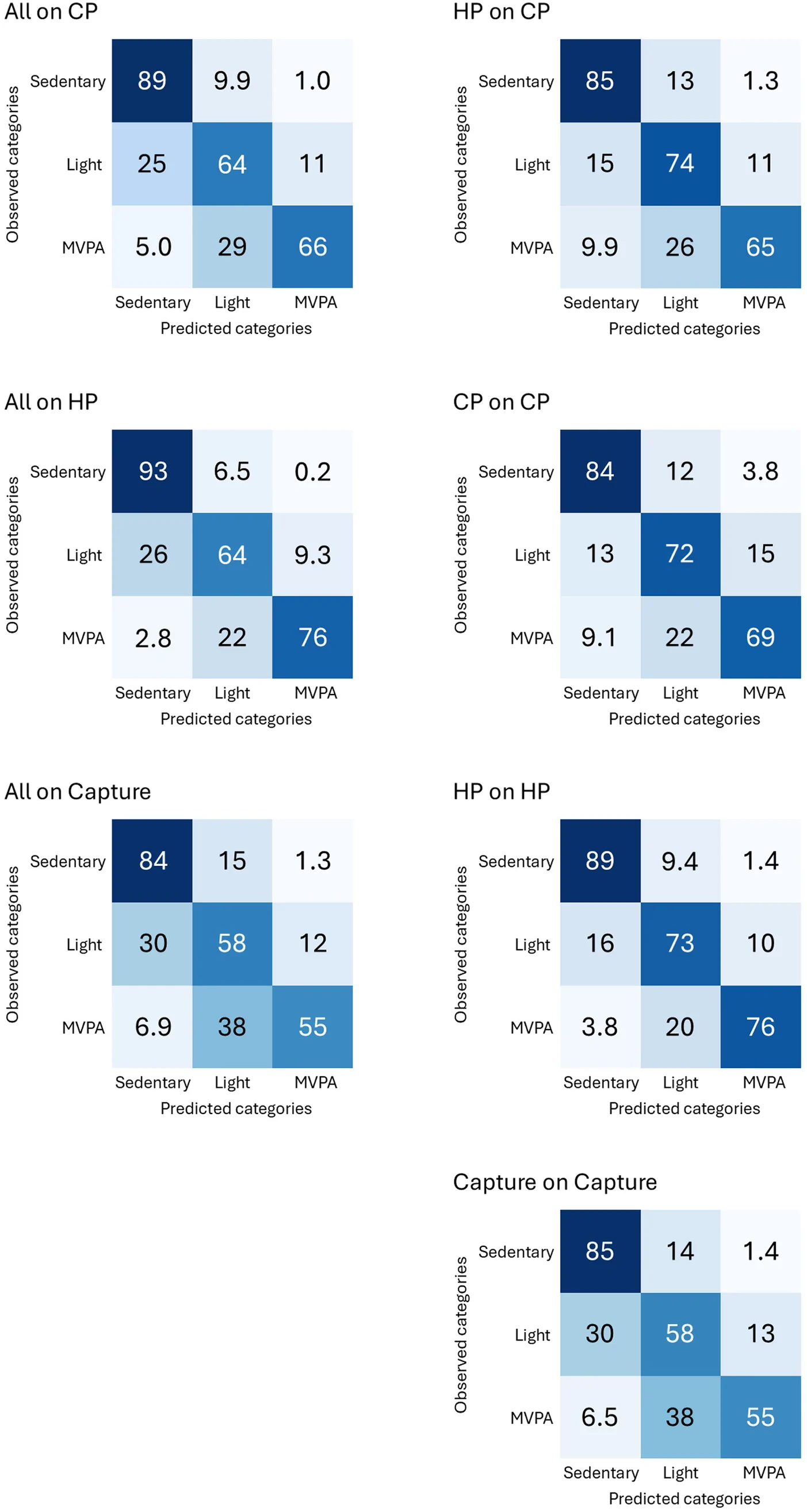
Confusion matrices illustrating the proportion of correctly and incorrectly classified activity intensity across the three classes, for each of the seven strategies.

Performance was comparable across the “All on CP”, “HP on CP”, and “CP on CP” strategies, suggesting that CP-specific training does not confer an advantage in our datasets.

### 3.3 Real-world construct validity

After the researchers individually assigned MET values to each recorded activity, inter-rater agreement for all minutes of activity intensity classification was 81%. All discrepancies were resolved through recalculation, discussion, or reconsideration of the most appropriate activity listed in the Adult Compendium of Physical Activities.

Table 4 presents the averaged individual agreement percentages of the DL model compared to the 1-minute observations, for participants with and without CP, and the weighted Cohen's kappa with linear weights. Agreement was 78.6% in HP and 73.6% in participants with CP. Individual agreement varied widely, ranging from 57.1% to 94.3%. Consistent with the results of training, testing and validating, agreement was lower for CP data, primarily due to the model's overclassification of sedentary activity (Figure 5) in 26% of cases. Misclassifications may stem from both observer judgment and model limitations. Weighted Cohen's kappa was 0.60 for CP and 0.66 for HP, which may be interpreted as moderate agreement for CP and substantial agreement for HP.

**Table 4:** Agreement (%) of activity intensity classification per minute (DL model vs. real-world observations) per individual, averaged over HP and CP participants separately, and weighted Cohen's kappa with linear weights.

CNN-model	Agreement (%)	Weighted Cohen's kappa
HP (*n* = 10) (SD) [range]	78.6 (9.6), [57.1, 94.3]	0.66
CP (*n* = 5) (SD) [range]	73.6 (12.0), [57.3, 87.2]	0.60

**Figure 5:**
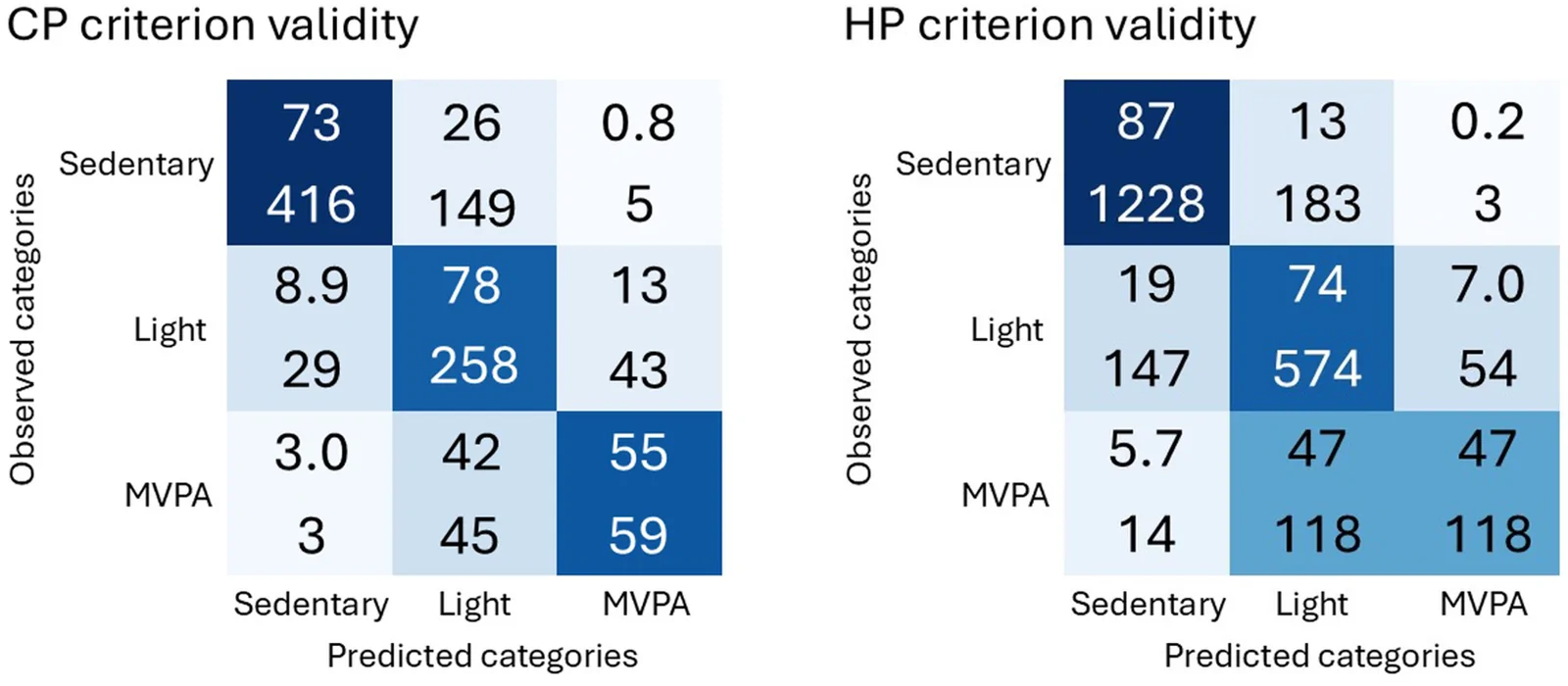
Confusion matrices illustrating the proportion and number of minutes with correctly and incorrectly classified activity intensity classes for the DL model vs. real-world observations, for CP and HP.

## 4 Discussion and conclusions

### 4.1 Results and comparison with previous studies

Patterns of activity and rest matter in CP rehabilitation. Pattern parameters can be extracted from accelerometer-derived time series of activity intensity. However, to obtain these patterns, a solid foundation of activity intensity recognition is needed. To date, algorithms that convert raw accelerometer data into activity intensity classes have not been trained, tested, and validated in CP populations, nor have they been evaluated in real-world settings. Therefore, we aimed to (1) develop a publicly available and directly applicable DL model to accurately classify activity intensity from raw tri-axial accelerometer data in CP populations, (2) investigate whether a separate DL model is needed for individuals with CP compared to HP and the general population, and (3) evaluate its real-world construct validity.

The DL model achieved good performance when trained on lab data from the 30 HP and 27 CP participants. While the addition of the large Capture-24 dataset improved output stability, it slightly reduced overall performance (Table 3). Importantly, all four DL models initially tested outperformed the conventional cut-points approach (Supplementary Appendix A), consistent with the conclusions of previous research in the general population (29). Performance values of the CNN model for recognizing three activity intensity classes were good and in accordance with previous DL studies in general populations (38, 55, 56). With strategies incorporating Capture data, light intensity activities and MVPA intensity activities were underestimated more frequently than in strategies that did not include Capture data. This might be explained by the relatively coarse labelling of observed activities of the Capture data, or by some inconsistencies in the Capture-24 classification of activities into intensity class, as 15 light intensity activities (MET > 1.5) were classified as sedentary activity, and 4 MVPA were classified as light intensity.

Downsampling Capture data from 100 to 12.5 Hz reduced the performance to some extent when trained and tested exclusively on Capture data (Supplementary Appendix A). This decline was more than compensated for by incorporating Lab data, which may indicate the relative importance of annotation precision. These results align with recent findings on the impact of sampling frequency on activity and intensity recognition performance (41, 42). When comparing frequencies of 10 Hz (41) or 13 Hz (42) with higher frequencies up to 100 Hz, the authors reported that reducing the frequency to 10 or 13 Hz did not lead to a substantial decline in accuracy of classifying activities and intensities. Consequently, using 10–13 Hz provides an effective balance between maintaining model performance and minimizing data volume.

Model performance on CP data was similar in magnitude for the model trained on the pooled dataset (Capture, HP, and CP data), the model trained on HP data, and the model trained exclusively on CP data. These findings do not indicate a clear advantage of a CP-specific algorithm over models trained on HP data or the pooled dataset within the populations included in this study. Despite potential differences in movement patterns (32, 57–59), these differences did not appear to affect activity-intensity classification performance in the datasets included in this study.

When evaluating real-world construct validity, agreement was higher in HP than in CP. Percentage of agreement was acceptable, and weighted Cohen's kappa was 0.60, at the boundary of moderate to substantial agreement (54). These real-world construct validity values cannot be directly compared with results from previous studies. While activity intensity classification algorithms have frequently been validated under controlled conditions, both their construct validity and criterion validity in free-living settings have received limited attention. Existing studies are scarce and primarily focus on classification performance, such as the ability to distinguish between activity types, rather than on the accuracy of minute-by-minute activity intensity estimates in daily life (60). This gap in the literature may reflect several methodological challenges, including the absence of a feasible and sufficiently accurate reference standard for ecological validation of activity intensity. Several of these challenges were also encountered in the present study.

A closer inspection of disagreement between the model's output and the comparator instrument suggests that discrepancies arose from multiple sources, including limitations of the comparator instrument, ambiguity in activity annotation, and individual differences in movement patterns. First, observer-based intensity classifications were sometimes inherently uncertain. This was particularly evident during minutes containing multiple activities, where assigning a single intensity category required averaging activities with different metabolic demands. Inter-observer agreement was low for these combined-activity minutes, which also showed low agreement between observations and DL classification. More generally, the comparator instrument relied on a predefined list of activities and corresponding MET values, irrespective of common individual variations in task execution. For example, the Adult Compendium of Physical Activities assigns sitting and speaking a MET value of 1.3 without distinguishing between speaking with or without gesticulation. When substantial arm movements occur during speech, the model may classify a higher activity intensity than the annotation suggests.

Second, several participants demonstrated systematic over- or underestimation across specific activities, suggesting the influence of individual movement characteristics. For example, 16 of 29 cycling minutes were underestimated for HP10, 103 of 120 min of sitting and talking were overestimated for CP5, and 22 of 38 min of walking at 3 km/h were overestimated for CP4. Such consistent patterns indicate that individual differences, rather than random classification errors reduced construct validity. This interpretation is supported by *post hoc* analyses of acceleration magnitude (Supplementary Appendix B). Two participants with chronic pain (CP4 and CP5), diagnosed with fibromyalgia, patellofemoral pain syndrome and neuroma, and fibromyalgia and non-specific low back pain respectively, showed markedly lower performance metrics and substantially higher acceleration magnitudes during sedentary and light-intensity activities than the rest of the sample. These findings may suggest elevated motor activity that was not captured by the annotation procedure, and may partly explain the model's overestimation relative to the comparator instrument.

Finally, activities located near the boundaries of the sedentary, light-intensity, and MVPA categories posed additional classification challenges. Small differences around the MET cut-off values of 1.5 and 3.0 can result in different intensity labels despite very similar movement patterns. For example, slow-paced walking (MET 2.8) was annotated as light intensity in CP4 and HP4, whereas the model classified 22 of 38 min as MVPA for CP4, and 26 of 41 min for HP4. Given the elevated acceleration magnitudes observed in these participants, these classifications appear physiologically plausible.

Taken together, additional investigation of real-world construct validity remains warranted, especially in CP populations. Moreover, before application of the model in research or clinical practice, other clinimetric properties, like reliability and responsiveness of outcome measures, should be investigated.

### 4.2 Strengths and limitations

To the best of our knowledge, this study is the first to evaluate the performance of a DL model for classifying three activity intensity classes from wrist-worn accelerometry in CP populations, and to analyze individual real-world construct validity. We implemented distinct datasets collected and annotated by independent parties, and we combined lab data and real-world data, which underlines the robustness of the results. Overall, the model demonstrated promising performance, but limitations of the current exploration of real-world construct validity warrants additional investigation, especially in CP populations, and at the individual level.

Some methodological aspects may introduce uncertainty regarding the results and their applicability at the individual level. First, limitations in the real-world observations likely led to underestimation of the true construct validity, while inter-individual differences in motor behavior may have added variability to model performance. Second, because performance varied substantially between individuals, particularly within the CP sample, the overall results may have differed if a different group of participants had been included.

Third, the potential influence of being observed must be acknowledged, as participants’ awareness of observation may have altered their usual activities and movement patterns. Fourth, the five individuals with CP included in the construct validity analysis differed from those in the CP lab data; they reported lower pain intensity and less disability. Individuals with more severe pain or functional limitations may display greater movement pattern deviations, which could affect model performance. Practical challenges associated with conducting real-world observations in people with CP limited our ability to recruit participants with a broader range of symptom severity.

Lastly, it is important to note that our work focuses specifically on the assessment of activity intensities. Although highly relevant in CP populations, we did not investigate the detection of specific activities or pain-related movement behavior such as guarding, avoiding and rubbing. A dedicated dataset and multiple studies on the topic of the detection of pain-related movement behavior are available (61, 62).

### 4.3 Recommendations

Before clinical or research application of the DL model it is recommended to further investigate construct validity, for example, in individuals with CP who exhibit pronounced differences in motor behavior. Moreover, factors such as hypervigilance, obesity, pain location, or the use of walking aids may influence individual model performance. A deeper understanding of these influences contributes to the development of robust and generalizable DL models tailored to the heterogeneity of CP patients. Construct validity can be further assessed with comparator instruments, and by investigating sensitivity to change (responsiveness). Moreover, reliability and measurement error should be investigated, and criterium validity in real-world may be investigated when an established and feasible gold standard is available.

Additionally, as accuracy during training, testing and validation on the Capture data was reduced from 71.8% to 67.4% by downsampling from 100 to 12.5 Hz, but improved to 76.5%–80.5% by incorporating Lab data, the influence of sampling frequency on CNN model performance remains uncertain. Further investigation of the importance of sampling frequency for performance within CP populations is warranted.

Exploring a continuous measure of activity intensity—rather than discrete intensity categories—may help to address borderline classification errors and enhance sensitivity to clinical change. Although the responsiveness of the current DL model still requires evaluation, a continuous measure may provide greater sensitivity in detecting clinically meaningful changes.

### 4.4 Conclusions

We developed a DL model using wrist-worn tri-axial accelerometer data collected from diverse populations and in a variety of real-world and laboratory settings. The data were annotated using two distinct methods by two independent parties. This diversity, combined with a rigorous evaluation of multiple DL models across different datasets and sampling frequencies provides valuable insights into how CP may influence model performance.

The DL model developed in this study demonstrated promising performance in the CP population. Furthermore, results of the different strategies with HP data, CP data and the pooled dataset indicate that, with our dataset, CP-specific training yields no clear advantage.

Nevertheless, inter-individual variability in agreement between real-world observations and model prediction highlights the potential value of further investigating construct validity within a larger sample of CP, and with more diversity*.*

## Data Availability

The datasets presented in this study can be found in online repositories. The names of the repository/repositories and accession number(s) can be found below: https://zenodo.org/records/17801499.

## Supplementary material

SUPPLEMENTARY APPENDIX A Evaluation of five initial models to select an architecture for further investigation.

SUPPLEMENTARY APPENDIX B Post hoc analysis of acceleration magnitude per activity intensity class in the healthy and chronic pain samples.
