# Supplementary material for: An open-source deep learning model for classifying real-world activity intensities in persons with chronic pain using a wrist-worn accelerometer
Source: Front Digit Health. 2026 Oct 14;8:1783507. doi: 10.3389/fdgth.2026.1783507 (PMC13713683; doi:10.3389/fdgth.2026.1783507)
Supplement: Supplementary file 1 [file Supplementaryfile1.docx]

## Appendix A

### Model evaluation

Five different models were evaluated: 1) a cut-off points model, serving as a baseline model 2) a four-layered CNN model; 3) A four-layered Convolutional Neural Network combined with Long Short-Term Memory (CNN-LSTM); 4) A three-layered LSTM model; and 5) a twelve-layered CNN model. The model architecture is described below. Additional details about model training, the resulting trained models and the accelerometer dataset collected in this study are publicly available on <https://zenodo.org/records/17801499>.

The models were trained, tested and validated with all data, (Lab data and Capture data). Model 2 - 5 were implemented in Python 3.12.7 with TensorFlow (2.20.0)/Keras (3.12.0) using the Sequential API. Each model received a fixed-length window of 125 consecutive time steps with 3 input channels per time step (input tensor shape 125 × 3, excluding the batch dimension) and produced a probability distribution over the three activity classes through a final dense layer with a softmax activation.

Unless stated otherwise, the following settings applied to every architecture:

Convolutional layers: one-dimensional convolutions (Conv1D) with a kernel size of 3, a stride of 1 and a ReLU activation. Layers using "valid" (i.e. no) padding shorten the sequence by two-time steps; layers using "same" padding preserve the sequence length.

Pooling layers: MaxPooling1D with a pool size of 2 and a stride of 2, halving the sequence length (rounded down) while leaving the number of feature maps unchanged.

Batch normalization: applied per feature map with the Keras defaults (momentum = 0.99, epsilon = 1e-3). Each batch normalization layer contributes 4 parameters per feature map, of which 2 (the scale γ and offset β) are trainable and 2 (the moving mean and moving variance) are non-trainable; these moving statistics account entirely for the difference between the total and trainable parameter counts reported for each model.

Regularization: dropout was applied only at the positions indicated below, with the rates specified per model. No L1/L2 weight penalties were used.

Initialization: Keras defaults, i.e. Glorot (Xavier) uniform for convolutional and dense kernels, orthogonal initialization for the LSTM recurrent kernels, and zero-initialized biases (the LSTM forget-gate bias is initialized to one). LSTM layers used the default tanh activation with a hard-sigmoid recurrent activation and no recurrent dropout.

Optimization: all models were compiled with the Adam optimizer using the Keras default hyperparameters (learning rate = 0.001, β1 = 0.9, β2 = 0.999, ε = 1e-7), a categorical cross-entropy loss on one-hot encoded labels, and classification accuracy as the monitored metric.

Training: at most 150 epochs with a mini-batch size of 64 windows, balanced class weights and early stopping, as described in detail in the next section.

The weights and compiled model files of each architecture were stored (.weights.h5 and .keras) so that the reported results can be reproduced without retraining. Reported accuracy and F1-scores are macro averaged over the three classes on the held-out test set.

**Training and evaluation procedure**

All data were partitioned at participant level to prevent data leakage. As a result, all windows belonging to a given participant were assigned to a single subset only, and no participant contributed data to more than one of the training, validation, or evaluation sets within a model fit.

Participants were assigned to two pools beforehand. The complete procedure below was repeated 3 times with different random draws. Within each repetition, the participants of the evaluation pool were shuffled and successively partitioned into five equally sized, mutually exclusive folds, each containing one fifth of the pool (rounded down); any participants left over after five folds had been drawn were not used as an evaluation fold in that repetition. Each fold served once as the held-out evaluation set.

For a given fold, the remaining participants of the training pool, i.e. all participants of that pool who were not part of the current evaluation fold, were shuffled and split at participant level into a training set containing four fifths and an internal validation set containing one fifth of these participants. The internal validation set was passed to Keras as validation data and was used exclusively to monitor training and to trigger early stopping; it was not used to compute the reported performance. Each architecture was therefore fitted 15 times in total. Before every fit, the model was reset to its initial state by reloading the weights that had been saved immediately after model construction, so that no information was carried over between folds or repetitions.

Class imbalance was addressed per fit with balanced class weights, computed on the training windows of that fit only with the scikit-learn function compute_class_weight (class_weight = "balanced"), which assigns each class a weight equal to the total number of training windows divided by the product of the number of classes and the number of training windows of that class; these weights were passed to the loss function through the class-weight argument of model.fit.

Each fit ran for a maximum of 150 epochs with a mini-batch size of 64 windows and reshuffling of the training windows before every epoch. Training was terminated early when the classification accuracy on the internal validation set had not improved for 15 consecutive epochs (Keras EarlyStopping with monitor = "val_accuracy" and patience = 15). Because the option to restore the best weights was disabled, the weights retained after training were those of the final epoch rather than those of the epoch with the highest validation accuracy. No learning-rate schedule and no other callbacks were used. After training, the model was evaluated on the held-out fold, which had taken no part in either training or early stopping. No random seed was fixed during training.

### Baseline cut-off points model

A baseline model was created using a Gaussian mixture model (GSM). The model estimates the probability of a data point belonging to a specific cluster. The input of the model was the acceleration magnitude, calculated via the formula: $\Sigma\sqrt{{ax}^{2}+ {ay}^{2}+ {az}^{2}}-1$. The ‘minus one’ was added to eliminate the effect of gravity. The clusters were defined as sedentary, light intensity and moderate-vigorous intensity. The results are visualized in figure A1. The GMM model achieved a moderate accuracy and F1 score of 59.5% and 51.5% respectively (table A1). The confusion matrix is presented in figure A2.

**Figure A1.** Histogram plot of the density of the summed acceleration magnitude per activity.

### CNN Model

The CNN model started with a Conv1D layer with 256 filters and a kernel size producing an output shape of (123, 256), followed by a MaxPooling1D layer to reduce the output dimensions to (61, 256). Batch normalization was applied after the pooling layer to stabilize the learning process. This pattern was repeated with decreasing filter sizes: a second, third and fourth Conv1D layer with 128 filters, 64 filters, 32 filters, followed by max pooling, and batch normalization. Next, the output was flattened into a 1D vector. This flattened output was then passed through a dense layer with 32 units, followed by a dropout layer to mitigate overfitting. Finally, the model generated classifications using a dense layer with three units, each representing one of the classes. The total number of parameters in the model was 138,979, with 138,019 being trainable. The CNN model achieved an acceptable accuracy and F1-score of 67.7% and 66.1% respectively (table A1). The confusion matrix is presented in figure A2.

All four convolutional layers used a kernel size of 3 with a stride of 1, a ReLU activation and valid padding, so that every convolution shortened the sequence by two time steps, and each max-pooling layer used a pool size and stride of 2. In full, the input window of 125 time steps by 3 channels entered a first Conv1D layer with 256 filters (2,560 parameters), giving a feature map of 123 by 256, which max pooling reduced to 61 by 256 before batch normalization (1,024 parameters). The second Conv1D layer with 128 filters (98,432 parameters) produced a feature map of 59 by 128, reduced by max pooling to 29 by 128 and followed by batch normalization (512 parameters). The third Conv1D layer with 64 filters (24,640 parameters) produced 27 by 64, reduced to 13 by 64 and normalized (256 parameters). The fourth Conv1D layer with 32 filters (6,176 parameters) produced 11 by 32, reduced to 5 by 32 and normalized (128 parameters). Flattening this final feature map yielded a vector of 160 elements, which was passed to a dense layer with 32 units and a ReLU activation (5,152 parameters), a dropout layer with a rate of 0.5, and finally the output layer with three units and a softmax activation (99 parameters). Summing these layers gives the reported total of 138,979 parameters, of which 960 are non-trainable, namely the moving means and variances of the four batch normalization layers.

### CNN-LSTM Model

The CNN-LSTM model combined convolutional layers with Long Short-Term Memory (LSTM) layers to capture both spatial and temporal features of the input data. The model started with three Conv1D layers, each followed by a MaxPooling1D and a Batch Normalization layer, similar to the CNN model, but with additional layers to capture complex features. After the final convolutional layer, a LSTM layer with 64 units was added to process the sequence of features extracted by the convolutional layers. This was followed by a second LSTM layer, which further refined the temporal dependencies. The output of the LSTM layers was flattened and passed through a dense layer with 32 units, followed by a dropout layer. The final output was produced by a dense layer with 3 units. The total number of parameters in this model was 157,763, with 157,315 being trainable. The CNN-LSTM model achieved acceptable accuracy and F1-score of 69,0% and 68,3% respectively (table A1). The confusion matrix is presented in figure A2.

The convolutional part consisted of seven Conv1D layers in total, all with a kernel size of 3, a stride of 1 and a ReLU activation. Three of these used valid padding and were each followed by max pooling (pool size and stride of 2) and batch normalization, while the remaining four used same padding and therefore preserved the sequence length. In full, the input window of 125 time steps by 3 channels entered a first Conv1D layer with 128 filters and valid padding (1,280 parameters), giving 123 by 128, which max pooling reduced to 61 by 128 before batch normalization (512 parameters). A same-padded Conv1D layer with 128 filters (49,280 parameters) preserved this shape, after which a valid-padded Conv1D layer with 64 filters (24,640 parameters) produced 59 by 64, reduced by max pooling to 29 by 64 and followed by batch normalization (256 parameters). A same-padded Conv1D layer with 64 filters (12,352 parameters) preserved this shape, and a valid-padded Conv1D layer with 32 filters (6,176 parameters) produced 27 by 32, reduced to 13 by 32 and normalized (128 parameters). A final same-padded Conv1D layer with 32 filters (3,104 parameters) left the shape unchanged, so that the recurrent part received a sequence of 13 time steps with 32 features. The first LSTM layer with 64 units returned the full sequence (return_sequences = True; 24,832 parameters), giving 13 by 64, and the second LSTM layer, also with 64 units, returned only the final hidden state (33,024 parameters), giving a vector of 64 elements.

The subsequent flatten operation therefore acts on an already one-dimensional vector and leaves it unchanged. This vector was passed to a dense layer with 32 units and a ReLU activation (2,080 parameters), a dropout layer with a rate of 0.5, and the output layer with three units and a softmax activation (99 parameters). Summing these layers gives the reported total of 157,763 parameters, of which 448 are the non-trainable moving statistics of the three batch normalization layers.

### LSTM Model

LSTM models capture temporal dependencies within the sequential data using recurrent layers. The model started with a Bidirectional LSTM layer, which processes the input in both forward and backward directions, resulting in an output shape of (125, 256). Batch normalization was applied to the output of this layer to improve training stability, followed by a dropout layer to prevent overfitting. This was followed by a second LSTM layer with 64 units that further processes the sequence. The final LSTM layer reduced the sequence to a 32-unit vector, which was then passed through a dense layer with 32 units. After another dropout layer, the model outputs its classification through a dense layer with 3 units. This model had a total of 232,323 parameters, with 231,619 being trainable. The LSTM model achieved acceptable accuracy and F1-score of 69,3% and 68,1% respectively (table A1). The confusion matrix is presented in figure A2.

The bidirectional layer wrapped an LSTM with 128 units per direction, whose forward and backward outputs were concatenated into 256 features per time step (135,168 parameters), so that the input window of 125 time steps by 3 channels was mapped onto a sequence of 125 by 256. This layer was followed by batch normalization (1,024 parameters) and a dropout layer with a rate of 0.2. The second LSTM layer with 64 units also returned the full sequence (82,176 parameters), giving 125 by 64, and was likewise followed by batch normalization (256 parameters) and a dropout layer with a rate of 0.2. The third LSTM layer with 32 units returned only its final hidden state (12,416 parameters), giving a vector of 32 elements, again followed by batch normalization (128 parameters) and a dropout layer with a rate of 0.2. Because this vector is already one-dimensional, the subsequent flatten operation leaves it unchanged. It was passed to a dense layer with 32 units and a ReLU activation (1,056 parameters), a fourth dropout layer with a rate of 0.2, and the output layer with three units and a softmax activation (99 parameters). Summing these layers gives the reported total of 232,323 parameters, of which 704 are the non-trainable moving statistics of the three batch normalization layers. No attention mechanism was used in the final model.

### CNN Large

The CNN-large model was similar to the CNN model described above, however with two additional convolutional layers, followed by batch normalization per filter size. This model had a total of 666,019 parameters, with 663,139 being trainable. The model achieved acceptable accuracy and F1-score of 68,8% and 67,7% respectively (table A1). The confusion matrix is presented in figure A2.

The model consisted of four convolutional blocks with 256, 128, 64 and 32 filters respectively, each block containing three Conv1D layers (kernel size 3, stride 1, ReLU) that were each followed by their own batch normalization layer. Within a block, the first two convolutions used same padding and therefore preserved the sequence length, whereas the third used valid padding and was the only one followed by max pooling (pool size and stride of 2). The model thus contained twelve convolutional and twelve batch normalization layers, i.e. eight convolutional layers more than the baseline CNN.

In full, the input window of 125 time steps by 3 channels entered the first block, in which two same-padded Conv1D layers with 256 filters (2,560 and 196,864 parameters, each followed by batch normalization with 1,024 parameters) preserved a sequence of 125 time steps, after which a valid-padded Conv1D layer with 256 filters (196,864 parameters) produced 123 by 256, reduced by max pooling to 61 by 256 and followed by batch normalization (1,024 parameters). The second block applied the same scheme with 128 filters: two same-padded convolutions (98,432 and 49,280 parameters, each with batch normalization of 512 parameters) preserved 61 time steps, a valid-padded convolution (49,280 parameters) produced 59 by 128, and max pooling and batch normalization (512 parameters) yielded 29 by 128. The third block used 64 filters: two same-padded convolutions (24,640 and 12,352 parameters, each with batch normalization of 256 parameters), a valid-padded convolution (12,352 parameters) producing 27 by 64, and max pooling with batch normalization (256 parameters) yielding 13 by 64. The fourth block used 32 filters: two same-padded convolutions (6,176 and 3,104 parameters, each with batch normalization of 128 parameters), a valid-padded convolution (3,104 parameters) producing 11 by 32, and max pooling with batch normalization (128 parameters) yielding 5 by 32.

Because the first two convolutions of each block preserve the sequence length, the feature-map sizes after each pooling stage are identical to those of the baseline CNN, and the classification head is likewise identical: flattening the final feature map yielded a vector of 160 elements, which was passed to a dense layer with 32 units and a ReLU activation (5,152 parameters), a dropout layer with a rate of 0.5, and the output layer with three units and a softmax activation (99 parameters). Summing these layers gives the reported total of 666,019 parameters, of which 2,880 are the non-trainable moving statistics of the twelve batch normalization layers.

**Table A1.** Performance values (%) for each model.

|  | **Accuracy** | **F1 score** | **Recall** | **Precision** |
| --- | --- | --- | --- | --- |
| Cut-off points | 59.5 | 51.5 | 67.0 | 54.0 |
| CNN (SD), [range] | 67.7 (1.2), [65.4,69.8] | 66.1 (1.2), [63.5,67.7] | 65.8 (1.2), [63.0,67.2] | 67.6 (1.4), [65.6,69.4] |
| CNN-LSTM (SD), [range] | 69.0 (2.4), [63.3,72.2] | 68.3 (2.0), [63.9,70.9] | 67.9 (1.9), [63.6,70.1] | 69.6 (2.0), [65.2,72.8] |
| LSTM (SD), [range] | 69.3 (2.2), [65.2,72.6] | 68.1 (2.0), [64.4,70.8] | 68.1 (1.8), [64.5,70.4] | 69.1 (2.1), [64.3,72.5] |
| CNN LARGE (SD), [range] | 68.8 (1.5), [67.3,71.5] | 67.7 (1.5), [66.3,71.4] | 67.2 (1.5), [65.9,70.8] | 69.3 (1.7), [66.9,72.5] |

| Baseline   | CNN   | CNN large   |
| --- | --- | --- |
| LSTM   | CNN-LSTM   |  |

**Figure A2.** Confusion matrices per model. Observed categories are depicted on the y-axis. The predicted categories are depicted on the x-axis. The confusion matrices provide an indication of the true and false positives / negatives. A perfect model would score 100 (%) on the diagonal, while all other values are 0.

### Downsampling Capture data

To assess the effect of downsampling Capture data from 100Hz to 12.5Hz, the performance of the CNN-model was tested with both frequencies. Accuracy with 100Hz was 71.8%, and 67.4 % with 12.5Hz.

Table A2. Performance values of the CNN-model with 100 Hz Capture data and down-sampled Capture data.

|  | **Accuracy** | **F1 score** | **Recall** | **Precision** |
| --- | --- | --- | --- | --- |
| Capture on Capture 12.5 Hz (SD), [range] | 67.4 (1.8),  [62.4,71.1] | 66.2 (1.8),  [61.7,70.2] | 65.9 (1.8),  [62.0,69.7] | 67.9 (1.8),  [62.6,71.3] |
| Capture on Capture 100 Hz (SD), [range] | 71.8 (1.9),  [66.7,77.0] | 71.2 (1.9),  [66.6,75.5] | 71.4 (1.8),  [67.5,76.1] | 71.7 (1.9),  [66.6,75.7] |
