# Supplementary material for: An open-source deep learning model for classifying real-world activity intensities in persons with chronic pain using a wrist-worn accelerometer
Source: Front Digit Health. 2026 Oct 14;8:1783507. doi: 10.3389/fdgth.2026.1783507 (PMC13713683; doi:10.3389/fdgth.2026.1783507)
Supplement: Supplementary file 2 [file Supplementaryfile2.docx]

### Appendix B

### Post hoc analysis

This post hoc analysis was conducted to gain a better understanding of the accuracy differences within healthy participants and individuals with chronic pain. We calculated the average acceleration magnitude of the raw sensor signal for all participants who took part in the real-world measurement (Table B1). Although the acceleration magnitude itself was not directly used by the model, it can provide insight into potential anomalies in the data. For example, participants CP4 and CP5 showed higher-than-average values in the sedentary and light intensity categories, which likely led to discrepancies between observed and predicted values and consequently reduced their overall agreement with the comparator instrument.

Table B1. Average acceleration magnitude per activity intensity class, and agreement, per construct validity participant.

| **Group** | **Sedentary**  **Mean (SD), m/s^2^** | **Light intensity**  **Mean (SD), m/s^2^** | **Moderate-vigorous intensity**  **Mean (SD), m/s^2^** | **Agreement**  **%** |
| --- | --- | --- | --- | --- |
| CP1 | 3.1 (1.0) | 11.5 (4.4) | 6.1 (0.4) | 87 |
| CP2 | 3.8 (1.9) | 11.5 (4.6) | 16.5 (6.0) | 74 |
| CP3 | 4.1 (1.9) | 11.6 (4.5) | 21.7 (6.0) | 82 |
| CP4 | 6.1 (4.1) | 19.5 (5.8) | 24.5 (0.4) | 67 |
| CP5 | 6.6 (3.7) | 15.5 (5.7) | 25.1 (4.2) | 57 |
| HP1 | 1.2 (0.8) | 11.9 (5.1) | - | 94 |
| HP2 | 4.6 (2.0) | 9.6 (3.8) | 7.5 (4.5) | 81 |
| HP3 | 2.1 (1.5) | 9.7 (7.1) | 23.5 (12.2) | 81 |
| HP4 | 3.8 (2.8) | 16.5 (8.4) | 22.6 (9.4) | 74 |
| HP5 | 3.8 (2.8) | 8.3 (3.2) | 17.4 (6.4) | 79 |
| HP6 | 3.0 (1.3) | 7.9 (3.7) | 22.4 (7.7) | 85 |
| HP7 | 2.0 (1.4) | 5.4 (3.5) | 21.3 (1.2) | 57 |
| HP8 | 3.5 (2.3) | 7.5 (4.7) | - | 73 |
| HP9 | 2.5 (1.6) | 6.0 (3.7) | - | 80 |
| HP10 | 2.3 (3.0) | 9.6 (4.3) | 13.6 (3.5) | 81 |
